# Production of Polyphenolic Natural Products by Bract-Derived Tissue Cultures of Three Medicinal *Tilia* spp.: A Comparative Untargeted Metabolomics Study

**DOI:** 10.3390/plants13101288

**Published:** 2024-05-07

**Authors:** Zsolt Szűcs, Zoltán Cziáky, László Volánszki, Csaba Máthé, Gábor Vasas, Sándor Gonda

**Affiliations:** 1 Department of Botany, Division of Pharmacognosy, University of Debrecen, Egyetem tér 1, 4032 Debrecen, Hungary; szucs.zsolt@science.unideb.hu (Z.S.); mathe.csaba@science.unideb.hu (C.M.); vasas.gabor@science.unideb.hu (G.V.); 2 Healthcare Industry Institute, University of Debrecen, Egyetem tér 1, 4032 Debrecen, Hungary; 3 Agricultural and Molecular Research and Service Institute, University of Nyíregyháza, Sóstói út 31/b, 4400 Nyíregyháza, Hungary; cziaky.zoltan@nye.hu; 4 Doctoral School of Pharmaceutical Sciences, University of Debrecen, Egyetem tér 1, 4032 Debrecen, Hungary; 5 Balaton Limnological Research Institute, HUN-REN (Hungarian Research Network), Klebelsberg K. u. 3, 8237 Tihany, Hungary

**Keywords:** linden, lime tree, plant tissue culture, natural products, in vitro culture

## Abstract

Medicinal plant tissue cultures are potential sources of bioactive compounds. In this study, we report the chemical characterization of the callus cultures of three medicinal *Tilia* spp. (*Tilia cordata*, *Tilia vulgaris* and *Tilia tomentosa*), along with the comparison to bracts and flowers of the same species. Our aim was to show that calli of *Tilia* spp. are good alternatives to the calli of *T. americana* for the production of polyphenols and are better sources of a subset of polyphenolic metabolites, compared to the original organs. Calli were initiated from young bracts and grown on woody plant medium containing 1 mg L^−1^ 2,4-D and 0.1 mg L^−1^ BAP. For chemical characterization, a quality-controlled untargeted metabolomics approach and the quantification of several bioactive compounds was performed with the use of LC-ESI-MS/MS. While bracts and flowers contained flavonoid glycosides (astragalin, isoquercitrin) as major polyphenols, calli of all species contained catechins, coumarins (fraxin, esculin and scopoletin) and flavane aglyca. *T. tomentosa* calli contained 5397 µg g DW^−1^ catechin, 201 µg g DW^−1^ esculin, 218 µg g DW^−1^ taxifolin and 273 µg g DW^−1^ eriodictyol, while calli from other species contained lower amounts. *T. cordata* and *T. tomentosa* flowers were rich in isoquercitrin, containing 8134 and 6385 µg g DW^−1^, respectively. The currently tested species contained many of the bioactive metabolites described from *T. americana*. The production of catechin was shown to be comparable to the most efficient tissue cultures reported. Flowers and bracts contained flavonoid glycosides, including tiliroside, resembling bioactive fractions of *T. americana*. In addition, untargeted metabolomics has shown fingerprint-like differences among species, highlighting possible chemotaxonomic and quality control applications, especially for bracts.

## 1. Introduction

Plant tissue cultures are initiated from young explants of plant tissues and have a variety of possible applications, including micropropagation and conservation of endangered species. Regarding tissue cultures of medicinal plants, one of the most frequently cited applications is their potential use in the production of specialized metabolites. Essentially, plant tissue cultures are generally considered alternative sources of bioactive compounds for pharmaceutical, cosmetic and dietary applications [1].

The first step in establishing tissue cultures typically involves the generation of callus—an undifferentiated mass of cells—using a balanced ratio of auxins and cytokinins [2]. Calli can be initiated from a wide variety of young tissues, although bracts, which are modified leaves associated with reproductive organs, have been infrequently used as explants in previous studies [3,4,5]. Since the hormones required for sustaining stable growth interfere with the biosynthesis and accumulation of specialized metabolites [6,7], comparative studies on tissue cultures and organs of whole plants can yield interesting results. In particular, the chemical pattern of calli can be markedly different from that of the organs of wild plants [8,9,10]. Although the specialized metabolite production of these tissue cultures can be increased via modulation of the medium composition or elicitation [11,12,13], the pattern of metabolites is primarily dictated by the tissue type.

The current study aimed to characterize the specialized metabolites of the tissue cultures of three medicinal *Tilia* spp. and compare them to the inflorescences gathered from trees growing under natural conditions. The inflorescence of *Tilia* species, typically harvested with the bracts, is commonly referred to as “lime flower”, “linden flower” or “*Tiliae flos*” in the phytomedical literature. Its traditional use in Eastern and Mediterranean Europe spans millennia [14,15]. Notably, *T. cordata* and *T. platyphyllos* are among the most popular components of herbal teas in Russia [16], reflecting their widespread use across Eurasia as a traditional herbal remedy, as well as a tea substitute or recreational beverage. The genus also includes a plant with promising anxiolytic properties: the Mexican *T. americana* L. is traditionally used to alleviate insomnia and related conditions. While positive in vivo results for *T. tomentosa* and *T. americana* regarding this indication have been reported in the literature [17], clinical trials on extracts of *Tilia* spp. remain absent.

The bioactive constituents likely responsible for these effects belong to polyphenolics of the phenylpropanoid (shikimate) pathway. The inflorescences contain high amounts of flavonoids (mostly in glycosidic form), catechins ((+)-catechin, (−)-epicatechin, procyanidin oligomers) and other phenylpropanoids (such as coumarins and cinnamic acid derivatives), as well as polysaccharides and essential oil [14,18,19,20,21]. However, chemical characterization of the tissue cultures within this genus remains relatively limited. Only a few reports have provided data on *T. americana* tissue cultures containing coumarins or flavonoids [22,23].

The aim of this study was the chemical characterization of the tissue cultures of medicinal *Tilia* species prevalent in the western part of Eurasia. As reference materials, bracts and flowers of the same species were characterized. The primary objectives were to show that (1) calli of *Tilia* spp. serve as viable alternatives to the more extensively studied tissue cultures of *T. americana* and (2) calli of *Tilia* spp. are better sources of a specific subset of polyphenolic metabolites compared to the original organs. The method of choice for chemical characterization was a combination of quality-controlled untargeted metabolomics and quantification of compounds thought to be responsible for the anxiolytic activity of *T. americana*. Both approaches were carried out using LC-ESI-MS.

## 2. Results

### 2.1. Tissue Culture Performance

At the typical density of seven explants per Petri dish (9 cm diameter), the used calli lines of *T. vulgaris*, *T. tomentosa* and *T. cordata* yielded 0.275 ± 0.042 g DW, 0.152 ± 0.053 g DW and 0.238 ± 0.124 g DW biomass at the end of the 28-day culture period.

### 2.2. Specialized Metabolites of Tissue Cultures and Organs of Tilia spp.

A set of specialized metabolites were tentatively identified to level “B” identification (Table 1) [24]. Several of these compounds (marked with Id. level “A” in Table 1) were successfully quantified using authentic standards after calibration and showing proper accuracy (Table S1).

The untargeted and subsequent targeted data-dependent MS/MS analyses resulted in 95 and 50 MS/MS consensus spectra, respectively, of which 39 and 16 were successfully annotated. Altogether, 55 and 68 compounds were annotated with >0.8 probability at the natural product class level and natural product pathway level, respectively (Table S2).

### 2.3. Chemical Differences between Tissue Types and Tilia Species

**Figure 1:**
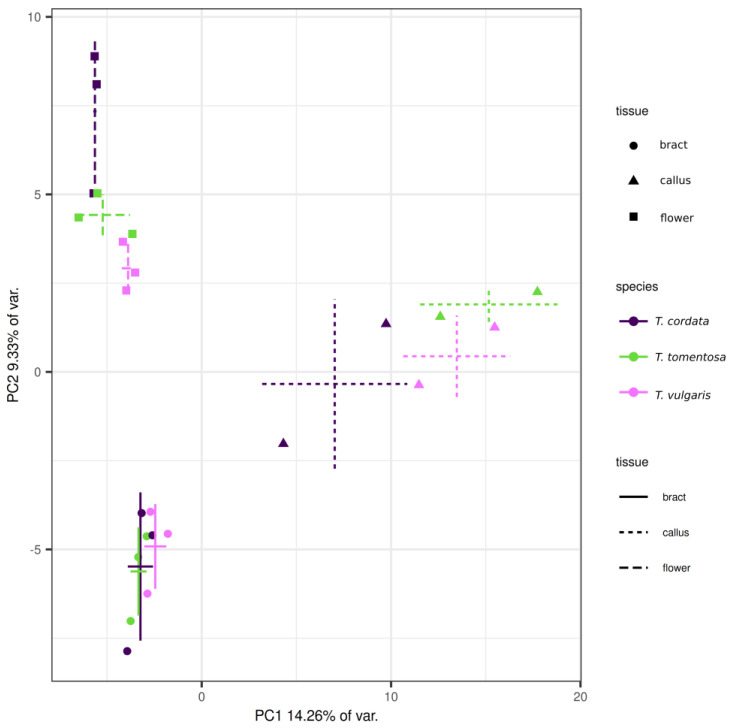
Principal component analysis biplot showing separation of various organs of different *Tilia* spp. according to their plant metabolome features. Axes show principal component order, with explained variance. Crosses denote average ± standard deviation for a species−organ pair (solid line, bract; dashed line, callus; long-dashed line, flower). Point shapes denote tissue type: circle, bract; triangle, callus; square: flower. Color denotes different *Tilia* species: purple, *T. cordata*; green, *T. tomentosa*; magenta, *T. vulgaris*.

**Figure 2:**
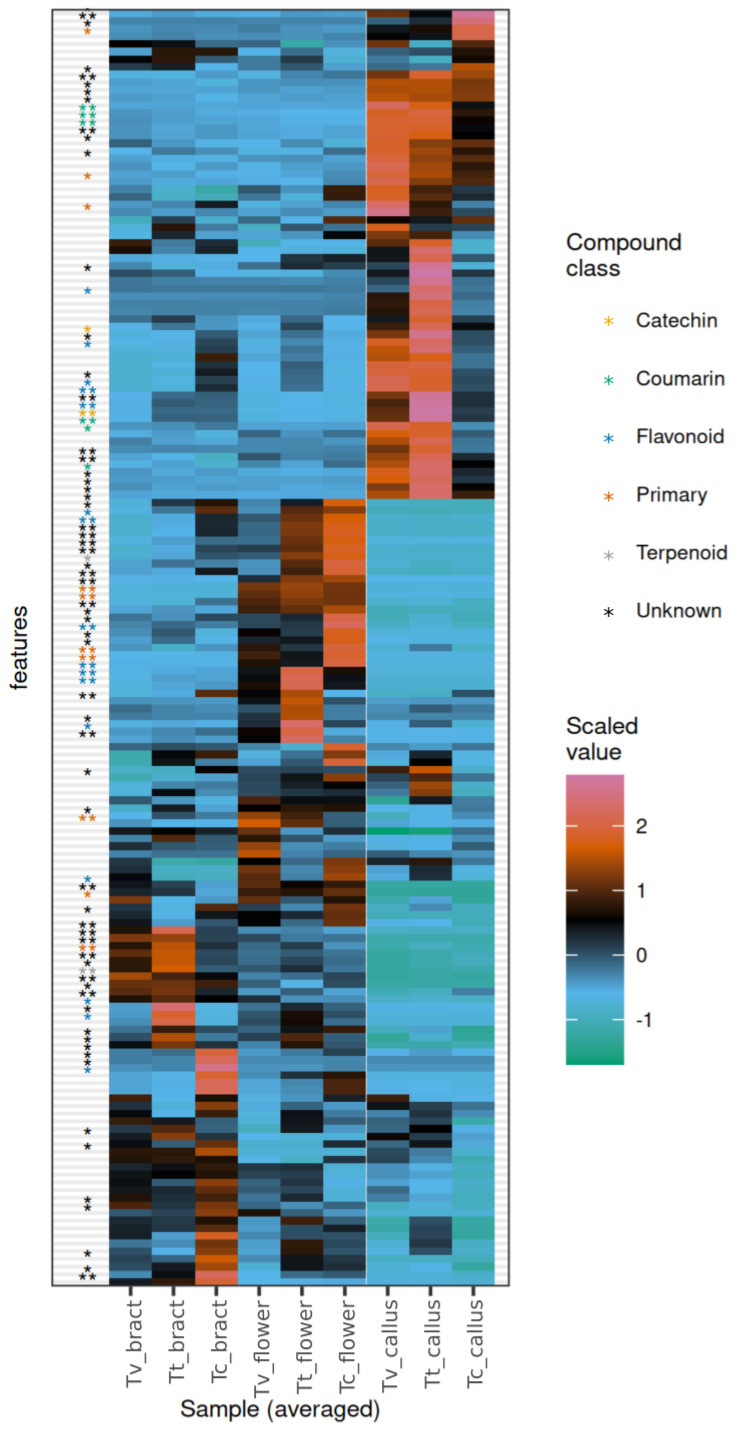
Heatmap showing relative abundance of chemical features in organs of *Tilia vulgaris*, *T. tomentosa* and *T. cordata*. The heatmap was generated from autoscaled, combined positive and negative ion mode data obtained through untargeted metabolomics using reverse phase LC-ESI-MS of the extracts of the organs. All features passed quality control filters. Species abbreviations: *Tc*, *Tilia cordata*; *Tt*, *Tilia tomentosa*, *Tv*, *Tilia vulgaris*. The asterisks to the left indicate the statistical significance of the Kruskal–Wallis test between species, after adjustment for false discovery rate: * *p* < 0.05; ** *p* < 0.01. The color of the asterisks corresponds to different compound classes, as shown in the legend (according to the annotation in SIRIUS).

The combined positive and negative ion mode datasets from untargeted metabolomics contained 167 features after the application of quality control filters. The differences observed between tissue types surpassed those observed within a single tissue type across species (Figure 1, Figure 2 and Figure S1). This was well supported by the significant differences in the principal components (PCs) between observation groups. PC1–4 together covered 38.54% of the variance of the dataset. PC1 and PC2 were significantly different among tissue types (Scheirer—Ray—Hare test, *p* = 7.94 × 10^−5^ and 0.00004, respectively), but not between species. PC4 was significantly different between species (*p* = 0.000326). Also, the species–tissue type interaction term was found to be significant for PC3 (*p* = 0.00274). Regarding individual features, a set of 98 features exhibited significant differences across tissue types (Table S2), with a false discovery rate (FDR) of 0.05. Flowers, bracts and calli exhibited distinct subsets of abundant features irrespective of species (Figure 2). However, upon closer examination, it becomes evident that all tissues from different species display a unique pattern of metabolites. The uniqueness of these patterns is especially apparent in the case of bracts (Figure 2), where several features are exclusive to a single species.

### 2.4. Coumarins

**Figure 3:**
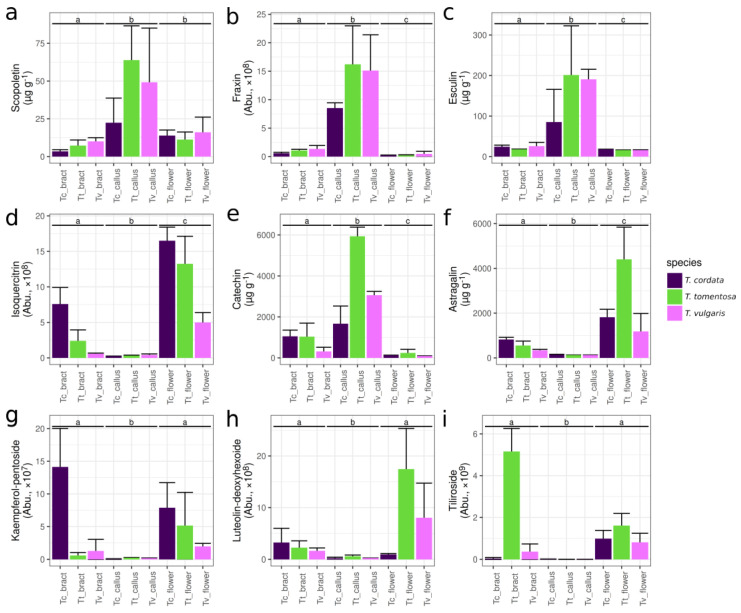
Concentrations or relative abundances of key bioactive constituents from various organs of *Tilia* species. Where an authentic standard was available, µg g DW^−1^ (dry weight) was given, in other cases, abundance (abbreviated “Abu.”) is shown. Subplots: (**a**) scopoletin; (**b**) fraxin (369.0819@7.77); (**c**) esculin; (**d**) isoquercitrin; (**e**) catechin; (**f**) astragalin; (**g**) kaempferol-O-pentoside (417.0821@8.43); (**h**) luteolin-O-deoxyhexose (431.0975@8.50); (**i**) tiliroside (595.1444@8.75). Species abbreviations on the *x* axis: *Tc*, *Tilia cordata*; *Tt*, *Tilia tomentosa*, *Tv*, *Tilia vulgaris*. Organs not sharing the same letter are significantly different at *p* < 0.05 (Dunn’s test, carried out only after significant Kruskal–Wallis tests adjusted for false discovery rate of 0.05).

Coumarins were relatively abundant in *T. vulgaris* and *T. tomentosa* calli, while they were present only in low to moderate amounts in other tested plant organs (Figure 3a–c and Figure S2a,b). Esculin was present in 191 and 201 µg g DW^−1^ in *T. vulgaris* and *T. tomentosa* calli, respectively, while bracts and flowers showed amounts ranging only from 16 to 26 µg g DW^−1^ (Figure 3c). A similar pattern was observed for scopoletin and fraxin (Figure 3a,b), as well as a methyl esculetin derivative (Figure S2b) and a putative scolopetin-O-hexoside (Figure S2a). The calli of *T. tomentosa* and *T. vulgaris* contained higher levels of coumarins than the calli of *T. cordata* (Figure 3a–c and Figure S2a,b). Overall, the six putative coumarins were present at 5.04–19.64-fold higher abundance in calli compared to bracts of the respective species (median values). Five of these compounds exhibited significant differences between organs (*p*_adj_ < 0.05, Kruskal–Wallis test).

### 2.5. Catechin Derivatives

Catechin derivatives emerged as another characteristic compound group in calli; however, the raw abundance data showed a mixed distribution among sample types. Catechin was an abundant member of this group, with concentrations ranging from 1667 to 5937 µg g DW^−1^ in the calli of various species (Figure 3e). This is 13.36- to 32.37-fold and 1.58- to 9.61-fold more than the amount found in flowers and bracts of the respective species, respectively. Another compound with an identical MS/MS spectrum (possibly epicatechin) displayed a similar pattern across the dataset, whereas two oligomeric catechins (putative proanthocyanidins) and a putative gallocatechin demonstrated much less variability. In these cases, calli and other tissues showed comparable levels of these compounds (Figure S2c,d).

Differences were also observed among the tested species. While *T. tomentosa* and *T. vulgaris* calli were rich in catechin-like compounds, *T. cordata* calli contained roughly the same amounts as the bracts. Specifically, in *T. tomentosa* and *T. vulgaris*, a median 8.67-fold and 11.26-fold higher abundance was shown compared to the bracts, respectively, whereas *T. cordata* showed a median fold change of only 0.96 (n = 9 putative catechin derivatives from untargeted screening). Gallocatechin was detected only in trace amounts in *T. cordata* flowers.

### 2.6. Flavonoid Glycosides

Flavonoid glycosides were present in relatively low amounts in the calli of *Tilia* spp. (Figure 3d,f–i and Figure S2e), despite these compounds being chief constituents of medicinal *Tilia* flowers and bracts. In the untargeted metabolomics dataset examined by CANOPUS, nine features appeared as flavonoids with >0.9 probability. The most probable level 5 ClassyFire class for most of these compounds was “flavonoid-O-glycosides”. These compounds behaved similarly: in the flowers of *T. cordata*, *T. tomentosa* and *T. vulgaris*, flavonoid glycosides were detected at 70.81-fold, 42.00-fold and 34.61-fold higher abundances, respectively, compared to calli (median values).

Flowers and bracts contained high amounts of isoquercitrin (=quercetin-3-O-glucoside) and astragalin (=kaempferol-3-O-glucoside), ranging from 1673 to 8134 µg g DW^−1^ and 1183 to 4400 µg g DW^−1^, respectively, depending on species (Figure 3d,f). Calli contained 19.41- to 66.08-fold less flavonoids than the flowers of their respective species for the three studied *Tilia* spp. (median of fold-change compounds, n = 13). Nonetheless, the tested calli still contained 6.07- to 7.20-fold higher levels of quercetin-3-O-glucoside than previously reported for the cell suspensions of *T. americana* [23].

Tiliroside (Figure 3i), a special phenylpropanoid conjugate of a kaempferol glycoside (kaempferol-3-β-D-(6″-p-coumaroyl-glucopyranoside)), was undetectable in the calli. In our dataset, the comparison of abundances to the similar isoquercitrin (quantified with an authentic standard) suggests that tiliroside is a relatively minor compound, with the exception of *T. tomentosa* bracts. Another kaempferol glycoside, a kaempferol-O-pentoside, was also biosynthesized in calli. However, only trace amounts were detected compared to flowers (Figure 3g).

Additionally, a luteolin-O-deoxyhexose was found in substantial amounts in *T. tomentosa* and *T. vulgaris* flowers, and to a much lesser extent in bracts, but none of the calli biosynthesized comparable amounts (Figure S2e).

### 2.7. Flavonoid Aglyca

Two flavonol aglyca with a wide distribution among plants are quercetin and kaempferol. These were found to be minor constituents compared to glycosides in all organs and showed no major variability between organs or species (Figure S2f,g). The concentration range for kaempferol and quercetin in natural tissues ranged from 12.84 to 36.03 µg g DW^−1^ and 16.17–40.79 µg g DW^−1^, respectively. Calli contained the same orders of magnitude of these compounds, with no significant difference observed between organs.

In contrast, the flavane aglycon eriodictyol and the flavanol aglycon taxifolin exhibited accumulation patterns similar to that of catechin, which also has a saturated C ring. In particular, *T. tomentosa* and *T. vulgaris* calli accumulated significant amounts of these compounds, while other tissues did not. Compared to other tissues of the respective species, *T. tomentosa* calli contained 16.6- to 19.2-fold more eriodictyol and 23.9- to 24.4-fold more taxifolin, while *T. vulgaris* calli contained 8.54- to 8.56-fold more eriodictyol and 10.0- to 12.8-fold more taxifolin. *T. tomentosa* calli accumulated most of the above compounds, with flavonoid concentrations ranging from 218 to 273 µg g DW^−1^.

## 3. Discussion

### 3.1. Coumarins from Organs and Tissue Cultures of Tilia spp.

Coumarins have previously been reported in various organs of trees in the genus *Tilia*. In this study, these compounds were identified based on MS/MS data of the published literature [25,26,27]. Fraxin, for instance, has been documented in the trunks of *T. amurensis* [32], the bracts of *T. platyphyllos* [21] and the tissue cultures of *T. americana* [33]. Similarly, scopoletin has been detected in the flowers of *T. cordata*, the trunks and bark of *T. amurensis* [32,34,35] and in tissue cultures of *T. americana* [33,36].

The current study has shown that 85–201 µg g DW^−1^ esculin and 22–64 µg g DW^−1^ scopoletin is present in calli (Figure 3a,c), while bracts and flowers are characterized by lower concentrations. One of our key objectives was to compare the tissue cultures to those of the anxiolytic *T. americana*. Unfortunately, available data for quantification of bioactives in calli and cell suspension cultures of *T. americana* were limited to data on scopoletin content [33,36]. Importantly, the amount of scopoletin in the calli of *T. tomentosa* and *T. vulgaris* was 2.22-fold and 1.71-fold higher, respectively, than that reported for a copper-elicited *T. americana* cell suspension [36]. Other coumarins such as esculin were present at more than 6-fold concentrations, indicating that tissue cultures of common medicinal *Tilia* spp., especially those of *T. tomentosa*, could serve as better sources of coumarins than tissue cultures of *T. americana*. It is important to note that the values presented in this study are before further optimization for the production of bioactive compounds.

Several studies have come to a conclusion supporting our findings, namely that some in vitro tissue cultures can generate significantly more coumarins than the intact plants [37,38]. However, when comparing our results to the most efficient coumarin-producing tissue cultures reported in the literature, the productivity of *Tilia* calli cannot be considered exceptional. For instance, under optimized conditions, calli of *Operculina turpethum* (L.) Silva Manso were reported to contain 5.3 ± 0.01 mg g^−1^ DW of coumarin [39]. Similar concentrations of scopoletin were observed in calli of *Solanum virginianum* L. (syn. *Solanum xanthocarpum* Schrad) [40,41] and *Eclipta prostrata* (L.) L. (syn. *Eclipta alba* (L.) Hassk.) [42]. It is worth noting that cultures with similar biosynthetic capacity have also been documented. For example, calli of *Ruta chalepensis* L. accumulated psoralen (370.12 ± 10.6 µg g^−1^ DW), along with lesser amounts of umbelliferone and xanthotoxin [43]. Similarly, the embryogenic calli of *Abutilon indicum* (L.) Sweet contained 99.20 ± 0.97 and 61.03 ± 0.47 μg g^−1^ FW scopoletin and scoparone [37].

### 3.2. Catechin Derivatives from Organs and Tissue Cultures of Tilia spp.

The compounds identified in this study included catechin, which was previously documented in *Tilia* inflorescences [20]. The high rate of catechin biosynthesis is also present in the original tissue from which calli are initiated: very young bract tissues of *Tilia platyphyllos* exhibit very high catechin content [21]. Interestingly, although catechins are present in even higher amounts compared to coumarins in *Tilia* calli, previous characterization of *T. americana* tissue cultures did not report any catechin-like compounds [23,33,36].

When compared to catechin-producing tissue cultures of other species, a surprising conclusion can be drawn: *Tilia* calli are among the most efficient in vitro producers of catechin reported to date. In the calli of the current study, 1667–5937 µg g DW^−1^ catechin was detected, which is significantly higher than the catechin content of most cell suspension cultures and calli reported for a variety of genera [8,44,45,46,47,48]. It must also be noted that the above studies mostly reported optimized production conditions. Only a few comparatively effective tissue cultures were reported: the same order of magnitude was observed for the elicited calli of *Thymus daenensis* Čelak. (1.99 mg g^−1^ DW) [49], the elicited calli of *Hypericum triquetrifolium* Turra (1.26–1.48 mg g DW^−1^ epicatechin) [50] and the calli of the best-known medicinal plant containing catechins, *Camellia sinensis* (L.) Kuntze, which contained approximately 4, 8 and 28 mg g DW^−1^ epicatechin, catechin and epigallocatechin, respectively [51]. Altogether, *Tilia* calli can be considered competitive alternatives to other calli in the production of catechins.

In contrast to our findings, two studies reported much higher catechin content in organs of wild plants compared to that in tissue cultures: a 4.8- to 18.5-fold advantage was found in *Clinacanthus nutans* (Burm.f.) Lindau [8], while a striking 11,930-fold difference was found in *Hypericum perforatum* L. [9]. This contrast highlights the importance of independently testing various genera.

### 3.3. Flavonoid Glycosides from Organs and Tissue Cultures of Tilia spp.

We detected several flavonoids and their glycosides that have been previously found in various *Tilia* spp. [20,28,52,53]. Flavonoid glycosides are the chief constituents of the inflorescences of medicinal *Tilia* spp., exemplified by the high values for individual flavonoids in our dataset (Figure 3d,f). Nevertheless, the overall perspective shows a mostly downregulated flavonoid glycoside biosynthetic pathway in calli (Figure 2, Figure 3d,f–i and Figure S1e) compared to flowers and bracts, with individual flavonoid glycosides being present at a maximum of about 480 µg g DW^−1^ (Figure 3d,f–i and Table S3). This observation is comparable to data on other tissue cultures capable of producing flavonoid glycosides. For instance, a cell suspension culture of *Orostachys cartilaginea* Boriss. accumulated 608.8 μg g DW^−1^ quercetin 3-O-glucoside and 2229.4 μg g DW^−1^ kaempferol-3-rutinoside under optimal aeration conditions in a bioreactor [54]. Another study reported that adventitious root cultures of *Valeriana jatamansi* Jones ex Roxb. accumulated 451.85 µg g DW^−1^ total phenolic compounds, predominantly composed of kaempferol and rutin [55]. Rutin was also described in elicited cell suspension cultures of *Momordica charantia* L. at a concentration of 352 μg g DW^−1^ [56].

The downregulation of flavonoid glycoside biosynthesis is supported by data on *T. americana* [23,33,36]. In another study on cotton [57], where tissue cultures of various stages of differentiation were assessed for flavonoids, an upregulated flavonoid biosynthesis was observed during embryogenesis. Altogether, the level of differentiation may be a key contributor to the downregulated biosynthesis of flavonoid glycosides.

A deeper insight can be obtained from comparative studies in which both tissue cultures and organs of wild plants were examined. These studies have yielded mixed results, underscoring the need for empirical, case-by-case studies. Two contrasting examples shall serve to highlight this variability. In the case of *Petroselinum crispum* (Mill.) Fuss, cell suspensions accumulated apigenin and kaempferol at 0.01 and 0.02 mg g DW^−1^, while embryogenic cultures produced 0.32 and 0.2 mg g DW^−1^ of the same compounds. However, these values were dwarfed by the levels detected in the leaves: 1.8 and 1.0 mg g DW^−1^, respectively [10]. Conversely, a study on *Clinacanthus nutans* (Burm.f.) Lindau found comparable levels of luteolin and kaempferol between suspension cells and one of the tested organs [8].

One of the most important flavonoid glycosides in *Tilia* is tiliroside, since it is thought to contribute to the anxiolytic activity of *T. americana* extracts [58] and is biosynthesized by only a few genera. Interestingly, studies have yielded conflicting findings regarding the biosynthesis of tiliroside in *Tilia* tissue cultures. Tiliroside was described in *T. americana* calli at levels comparable to those found in leaves, but only in calli derived from apical buds; calli from other tested explants contained no tiliroside [23]. Another study on *T. americana var. mexicana* showed that tiliroside is not produced in in vitro cultures [36]. This finding is reinforced by another report [33], in which preparative purification of the extracts *T. americana* calli only yielded coumarins, triterpenes and a flavonoid glycoside. This inconsistency in tiliroside biosynthesis in *Tilia* tissue cultures may be the result of high strain-to-strain variability. Only two reports have documented biosynthesis of tiliroside in tissue cultures from other genera: Calli of *Chorisia* spp. [59] and *Paratecoma peroba* (Record) Kuhlm [60]. The former reported values around 0.547 mg g DW^−1^. Altogether, while *Tilia* tissue cultures do not seem to be potential sources of tiliroside, further investigation of *T. tomentosa* bracts is certainly warranted, given their potential as candidates for testing anxiolytic activity.

### 3.4. Flavonoid Aglyca from Organs and Tissue Cultures of Tilia spp.

Quercetin and kaempferol also likely contribute to the anxiolytic activity of *T. americana* [58]. However, given their low levels in all organs tested in this study, their significance should not be overestimated. Moreover, several tissue cultures with much higher levels of these compounds have been reported in the literature. For instance, in vitro callus cultures of *Lepidium sativum* L. were found to contain 22.08 mg g DW^−1^ quercetin and 7.77 mg g DW^−1^ kaempferol under optimized conditions in a study testing various light conditions [61]. In another study, 6.19 mg g^−1^ DW quercetin and 5.48 mg g^−1^ DW kaempferol were produced by the calli of *Ipomoea turbinata* Lagasca under optimized conditions with thidiazuron–elicitation [62]. These findings obviously render *Tilia* calli less competitive as potential industrial producers of widely known flavonoid aglyca.

On the other hand, significantly less data are available on less common flavonoids with a saturated C-ring, such as eriodictyol and taxifolin, which were identified in our dataset. Our measurements indicated that *T. tomentosa* calli accumulated 273 and 218 µg g DW^−1^ of these compounds on average, respectively (Figure S2h,i). Concerning taxifolin production, the potency of calli from *Tilia* spp. were comparable to that of the suspension cultures of *Cascabela thevetia* (L.) Lippold (syn. *Thevetia peruviana* K.Schum.) [63] but fell short of the tissue cultures of *Larix gmelinii var. olgensis* Ostenf. & Syrach (syn. *Larix olgensis* A. Henry), which produced 4.77 mg g DW^−1^ taxifolin when treated with the optimal hormone combination [64]. However, there is a gap in the literature when it comes to data on well-proven eriodictyol-producing tissue cultures.

### 3.5. Unique Patterns in Untargeted Metabolomics Data

In studies using both untargeted metabolomics and quantification for comparative analysis of plant materials, the set of targeted compounds can be significantly increased, as demonstrated by Monti et al. [65]. In our study, despite the high variance between tissue types, discernable differences between organs of the three *Tilia* species emerged (Figure S1), revealing unique fingerprint-like patterns that could potentially enable source identification (Figure 2). Key differentiating natural products include flavonoids, as well as primary metabolites. The phenomenon was most apparent in bracts.

Potential applications of these unique fingerprints include identification, classification and chemotaxonomic evaluation of plant species, as well as quality control and authentication of industrial raw materials [66]. For example, Afzan et al. [67] effectively separated three chemotypes of *Ficus deltoidea* Jack varieties using a similar approach. Another study successfully utilized untargeted metabolomics to obtain chemotaxonomic information on three *Riccia* spp. [68]. The latter study was able to present a clear separation of the three species since chemical differences between species were greater than observed in our study. Moreover, Mannochio-Russo et al. [69] demonstrated the effectiveness of this approach at higher taxonomic levels, namely, across an entire plant family (Malpighiaceae). On the other hand, within-species examinations such as comparison of cultivars may yield a continuous gradient of points rather than distinct clusters [65].

Our data show that various tissue types contain highly distinct chemical patterns, as well as additional patterns that would have been overlooked had our analysis been restricted to compounds for which an authentic standard was available. Essentially, the fingerprints derived from quality-controlled untargeted metabolomics serve as valuable complements to the data on quantified natural products.

## 4. Materials and Methods

### 4.1. Chemicals

All reagents used were of at least analytical quality. The standards scopoletin, (+)-catechin, quercetin, kaempferol, taxifolin, eriodictyol, astragalin, isoquercitrin and esculin, as well as methanol, were procured from Merck/Sigma Aldrich (Rahway, NJ, USA). LC-MS grade acetonitrile, water and formic acid were purchased from Fisher Scientific (Geel, Belgium). Vendors for the components of the woody plant medium (WPM), hormones and vitamins are listed in the Supplementary Materials.

### 4.2. Plant Material

#### 4.2.1. Tissue Samples from Trees

*Tilia* species were identified based on specific morphological characteristics [70]. Biological replicates were obtained by collecting bract and flower samples from three adjacent trees of *Tilia cordata* Mill., *Tilia vulgaris* Hayne and *Tilia tomentosa* Moench (Malvaceae). The organs were collected from the campus of the University of Debrecen (47.5556 N, 21.6215 E) during the flowering period, 52–54 days after the appearance of the bracts. During sampling, representative samples were collected from several points on the trees.

#### 4.2.2. Tissue Culture Initiation and General Protocols

Young bracts aged 1–4 days from various *Tilia* spp. obtained from the same site were selected as explants. The bracts were surface sterilized in 3% NaOCl with 0.1% Polysorbate 20 for 3 × 5 min, and then washed with autoclaved bidistilled water and subcultured to pH 5.8 autoclaved woody plant medium (WPM) [71] with 3% sucrose, 1% agar, 1 mg L^−1^ 2,4-D and 0.1 mg L^−1^ BAP for callus initiation.

Established calli were subcultured to the same medium composition every 28 days. This culture period enabled the harvesting of healthy calli of 1–2.5 cm size (Figure S3). Typically, 100–150 mg fresh weight of tissue was transferred during each subculture per 9 cm Petri dish [13], at a density of 7 explants per dish. To obtain biological replicates, bracts from different trees were used as explants, and two stable, independent lines for each species were selected for chemical characterization. The callus lines were maintained under a photoperiod of 14/10 h (22/18 ± 2 °C in light/darkness). For illumination, white, fluorescent light was used (50 µmol m^−2^s^−1^). For chemical analysis, 28-day-old calli were used (Figure S3). The chemical characterization experiments were started after the calli were sufficiently stable. To ensure stability, at least 6 subcultures were carried out before enrolling cultures into this step of the experiments.

### 4.3. Phytochemical Analysis

#### 4.3.1. Sample Preparation

The drying procedure was carried out according to previous protocols [13,21], with additional details provided in the Supplementary Materials. Subsequently, the dried samples were stored in sealed vials, in darkness, at room temperature until further processing. The dried calli lines, as well as the bract and flower samples used in the study, were deposited in the Department of Botany, University of Debrecen.

Following the homogenization and extraction procedure already described [21], the samples were diluted 10-fold with MeOH and filtered through a 0.22 μm PTFE syringe filter prior to analysis. Process blanks, using identical protocols but without a plant matrix added, were employed as blanks for liquid chromatography–electrospray–mass spectrometry (LC-ESI-MS) measurement [72].

#### 4.3.2. LC-ESI-MS

The instrument and method described by Szűcs et al. [21] for the analysis of *Tilia* bracts were used with slight modifications. Details are provided in the Supplementary Materials.

#### 4.3.3. Method Performance Assessment

Method performance was assessed through the construction of 4–7-point calibration curves using authentic standards spanning 2–3 orders of magnitude. These curves were used to calculate linearity, lower limit of quantification (LLOQ) and upper limit of quantification (ULOQ). Intraday repeatability was assessed by calculating RSD values from the features of QC samples (a mixture of all MeOH extracts mixed at equal ratio, see below). Accuracy was calculated by spiking a 10-fold diluted pooled QC sample with 25% and 50% of the found concentration (n = 3). The results are shown in Table S1.

To obtain exact concentration data for a set of selected metabolites, the above calibration curves were used. Subsequently, a targeted quantitative assessment was performed in mzMine 2.53 [73], using parameters described in our recent study.

#### 4.3.4. Metabolite Annotation

MS/MS spectra were gathered from both untargeted and targeted measurements. The former was based on an exclusion list containing all features with intensities above 10^5^ (in either ion modes). This list was generated from a blank measurement using an in-house R script with mzR 2.30.0 and Spectra 1.6.0 [74]. The latter was based on a series of inclusion lists generated from features that passed all quality control metrics (see Section 4.3.5). The parameters are shown in Tables S4 and S5. Raw MS/MS spectra were merged into consensus spectra as outlined in [75] and annotated along the Classyfire and natural product class (NPC) hierarchies in SIRIUS 5.6.3 [76,77,78,79]. In parallel, a manual search was carried out based on the existing literature. These references were also used to manually verify SIRIUS suggestions in some instances.

Levels of identification, following the criteria outlined by Alseekh et al. [24], are defined as follows: (A) authentic standard enabled full identification; (B(i)) perfect match with a record in literature data; (B(ii)) MS/MS fragmentation deduced from schemes described in more generic literature and similar compounds in the current sample, with all major peaks explained; (B(iii)) the same as B(ii), but only partially explained spectra, leading to the description of derivatives of a well-defined moiety; (C) SIRIUS/CANOPUS class/superclass suggestions with >0.8 probability.

#### 4.3.5. Quality Controlled, Untargeted Metabolomics

Raw measurement files were converted to mzXML using mscovert and uploaded to XCMSOnline version 2.7.2 (XCMS version 1.47.3) for analysis [80]. Parameters are shown in Table S6. The resulting CSV files were processed in R, as outlined in our previous study [74].

Quality-controlled metabolomics measurements enable the drawing of quantitative conclusions regarding chemical features for which no authentic standards are available [72]. The “intra-study QC” approach described in our recent study [74] was used as a means of quality control. The data from the QC injections were used to filter features according to their linearity and precision and to apply LOESS readjustment of local inhomogeneities of sensitivity. Details are available in the Supplementary Materials.

### 4.4. Statistics

All statistical evaluations were carried out in R 4.3 [81]. Metabolite-level differences among tissue types were examined using the Kruskal–Wallis test, using the tissue type as the factor, followed by adjusting the *p*-values for false discovery rate (0.05) with the Benjamini–Yekutieli procedure, as in our recent paper [74]. The overall influence of the factors “tissue type” and “species” were assessed with the non-parametric Scheirer–Ray–Hare procedure. In brief, the scaled dataset was subjected to principal component analysis. Subsequently, PC1–4 were tested for significant differences along the factors “tissue type” and “species”.

## 5. Conclusions

This study aimed to evaluate the potential of tissue cultures from frequently used medicinal *Tilia* spp. as sources of polyphenolic bioactive natural products. Our chemical characterization approach utilized a combination of quality-controlled untargeted metabolomics and quantification of a set of important metabolites.

Bracts and flowers from the tested *Tilia* species contained flavonoid glycosides, including tiliroside, that are thought to be behind the activity of the anxiolytic *T. americana*, suggesting avenues for investigating similar effects in future research. Notably, metabolomics revealed unique patterns, particularly evident in various bract samples, highlighting the potential for further research in quality control or chemotaxonomy.

More importantly, our quantification results enabled us to conclude that the tested tissue cultures are potential alternative sources of catechins and flavonoid aglyca, and to a certain extent, coumarins, albeit not flavonoid glycosides. The most promising tissue cultures were the calli of *T. tomentosa*, which demonstrated particular efficiency in producing catechin, eriodictyol and, to some extent, taxifolin and coumarins.

## Figures and Tables

**Table 1:** Metabolites from flowers, bracts and calli of *Tilia cordata*, *T. tomentosa* and *T. vulgaris*, putatively identified at least at level B with LC-ESI-MS. Column header abbreviations: *m/z* (pos./neg.), mass-to-charge ratio of chemical features in positive or negative ion mode; Rt (min), retention time in minutes; *m/z* diff. ppm (pos/neg), mass-to-charge ratio difference from theoretically calculated value, expressed in ppm, in positive/negative ion mode; Id. level, identification level according to [24], see footer or Section 4.4. for more details; Ref., references.

Name	*m/z* (pos.)	*m/z* (neg.)	Rt (min)	Formula (M)	*m/z* diff. ppm (pos.)	*m/z* diff. ppm (neg.)	Major MS/MS Fragments	Id. Level	Ref.
Coumarins and derivatives									
Esculin		339.0725	7.45	C_15_H_16_O_9_		+2.6	177.0184; 133.0284	A	
Methylesculetin derivative		399.0936	7.67	NA		NA	191.0345 176.0106	B(iii)	[25,26]
Scopoletin-O-hexoside		353.0877	7.66	C_16_H_18_O_9_		+1.2	193.0499; 163.0391	B(i)	[27]
Fraxin		369.0830	7.77	C_16_H_18_O_10_		+2.2	207.0294; 192.0058; 163.0029	B(i)	[25,26]
Scopoletin	193.0493		8.38	C_10_H_8_O_4_	−4.0		161.0594; 133.0647; 105.0701	A	
Catechins and derivatives									
Gallocatechol		305.0670	7.52	C_15_H_14_O_7_		+2.9	261.0760; 219.0650; 179.0340; 167.0339; 139.0387; 125.02303	B(ii)	[20,28]
Catechin		289.0721	7.68	C_15_H_14_O_6_		+3.1	271.0621; 245.0813; 221.0811; 179.0338; 125.0231	A	
Catechin dimer		577.135	7.78	C_30_H_26_O_12_		+0.7	407.0766; 289.0716; 245.00814; 161.0230; 125.0229	B(i)	[28,29]
Catechin trimer		865.1990	7.92	C_45_H_38_O_18_		+1.2	407.0766; 289.0716; 245.0448; 125.0228	B(i)	[20,28]
Flavonoid glycosides									
Isoquercitrin		463.0887	8.18	C_21_H_20_O_12_		+2.3	301.0344; 300.0272; 271.0247; 151.0021	A	
Quercetin-malonyl-O- hexoside		549.0890	8.27	C_24_H_22_O_15_		+1.7	463.0889; 371.2072; 301.0356; 300.0279; 271.0254; 255.0298; 151.0024	B(ii)	[20,28]
Astragalin		447.0939	8.32	C_21_H_20_O_11_		+2.6	285.0408; 284.0332; 271.0255; 255.0298; 227.0351	A	
Quercetin-O-pentoside	435.0923		8.36	C_20_H_18_O_11_	−1.0		303.0498; 257.0447; 153.0182	B(i)	[20,28]
Kaempferol-O- pentoside		417.0828	8.41	C_20_H_18_O_10_		+1.5	285.0392; 255. 0293; 227.0341; 151.0022	B(ii)	[28,29]
Luteolin-O-deoxy- hexoside		431.0986	8.48	C_21_H_20_O_10_		+1.8	285.0409; 284.0391; 255.0297; 227.0343; 151.0024	B(i)	[20,28]
Tiliroside	595.1461		8.71	C_30_H_26_O_13_	+1.6		287.0894; 195.0294; 153.0184	B(ii)	[20,28]
Rhamnetin-O-hexoside		477.1039	8.74	C_22_H_22_O_12_		+1.3	315.0512; 301.0364; 271.0243	B(i)	[20,28]
Flavonoid aglyca									
Taxifolin		303.0504	8.36	C_15_H_12_O_7_		−0.3	287.0551; 259.0584; 231.0653; 153.01836	A	
Eriodictyol		287.0559	8.84	C_15_H_12_O_6_		+1.2	269.0458; 169.0129; 151.0022; 135.0437	A	
Quercetin		301.0349	8.89	C_15_H_10_O_7_		+0.2	273.0404; 255.1966; 151.002	A	
Trihydroxy-flavanone	273.0761		9.19	C_15_H_12_O_5_	−0.7		255.06561; 215.0465; 153.01836	B(ii)	[28,29]
Kaempferol	287.0554		9.23	C_15_H_10_O_6_	−0.4		255.0648; 215.0463; 187.0153; 153.0179	A	
Fatty acids									
Trihydroxy-linoleic acid		327.2180	9.03	C_18_H_32_O_5_		+2.6	309.2078; 291.1968; 229.1442; 211.1334; 183.1382; 171.1016	B(i)	[30]
Linoleic acid derivate		329.2336	9.2	NA		NA	293.2132; 229.1442; 211.1335; 183.1384; 171.1017	B(iii)	[30]
1-18:3-lysoPE	476.2765		10.35	C_23_H_42_NO_7_P	−2.5		335.2576; 304.2629; 261.2204	B(i)	[31]
1-18:2-lysoPE	478.2929		10.65	C_23_H_44_NO_7_P	−1.0		337.2735; 306.2791; 263.2369	B(i)	[31]
Dehydro-hydroxy- linoleic acid		293.2126	10.74	C_18_H_30_O_3_		+3.2	275.2020 235.1703 183.1381 171.1011	B(ii)	[30]
1-16:0-lysoPE	454.2932		10.94	C_21_H_44_NO_7_P	−0.4		313.2730; 282.2785; 239.2367; 155.0101	B(i)	[31]
Hydroxy-linoleic acid		295.2280	11.05	C_18_H_32_O_3_		+2.3	277.2174; 195.1386; 183.1386; 171.1016	B(ii)	[30]

Levels of identification are shown in column “Id. level” and defined according to Alseekh et al. [24] as follows: (A) authentic standard; (B(i)) confident match based on MS/MS; (B(ii)) confident match using in silico MS/MS approaches; (B(iii)) partial match based on MS/MS. For clarity, in the case of each compound, only the polarity chosen for fragmentation was shown, even if it was possible to detect the compound of interest in the other ion mode. Abbreviations: NA, not available.

## Data Availability

Data are available upon request.

## Supplementary Materials

The following supporting information can be downloaded at: https://www.mdpi.com/article/10.3390/plants13101288/s1, Additional results: Figure S1: Concentrations or relative abundances of key bioactive constituents from various organs of *Tilia* species. Figure S2: Principal component analysis biplot showing separation of various species of different *Tilia* spp. according to their plant metabolome features. Figure S3: Photos of stable callus cultures of tested *Tilia* species, at the end of their 28-day culture period, before harvesting. Table S1: Method performance parameters. Abbreviations: LLOQ, lower limit of quantification; RSD, relative standard deviation; ULOQ, upper limit of quantification. Table S2: Additional feature parameters. Table S3: Average concentrations of key bioactive constituents from various organs of *Tilia* species. Additional methodical information: More details are available for Sections S4.1 and S4.3.1–S4.3.5. Table S4: Data-dependent MS/MS parameters (ddMS) for targeted LC-MS/MS. Table S5: Data-dependent MS/MS parameters (ddMS) for untargeted LC-MS/MS. Table S6: XCMS Online automated peak search parameters. Table S7: Injection order for untargeted metabolomics.
