# Supplementary material for: Production of Polyphenolic Natural Products by Bract-Derived Tissue Cultures of Three Medicinal Tilia spp.: A Comparative Untargeted Metabolomics Study
Source: Plants (Basel). 2024 May 7;13(10):1288. doi: 10.3390/plants13101288 (PMC11124948; doi:10.3390/plants13101288)
Supplement: Supplementary file 1 [file plants-13-01288-s001.zip › SM.pdf]

Supplementary, Results and discussion

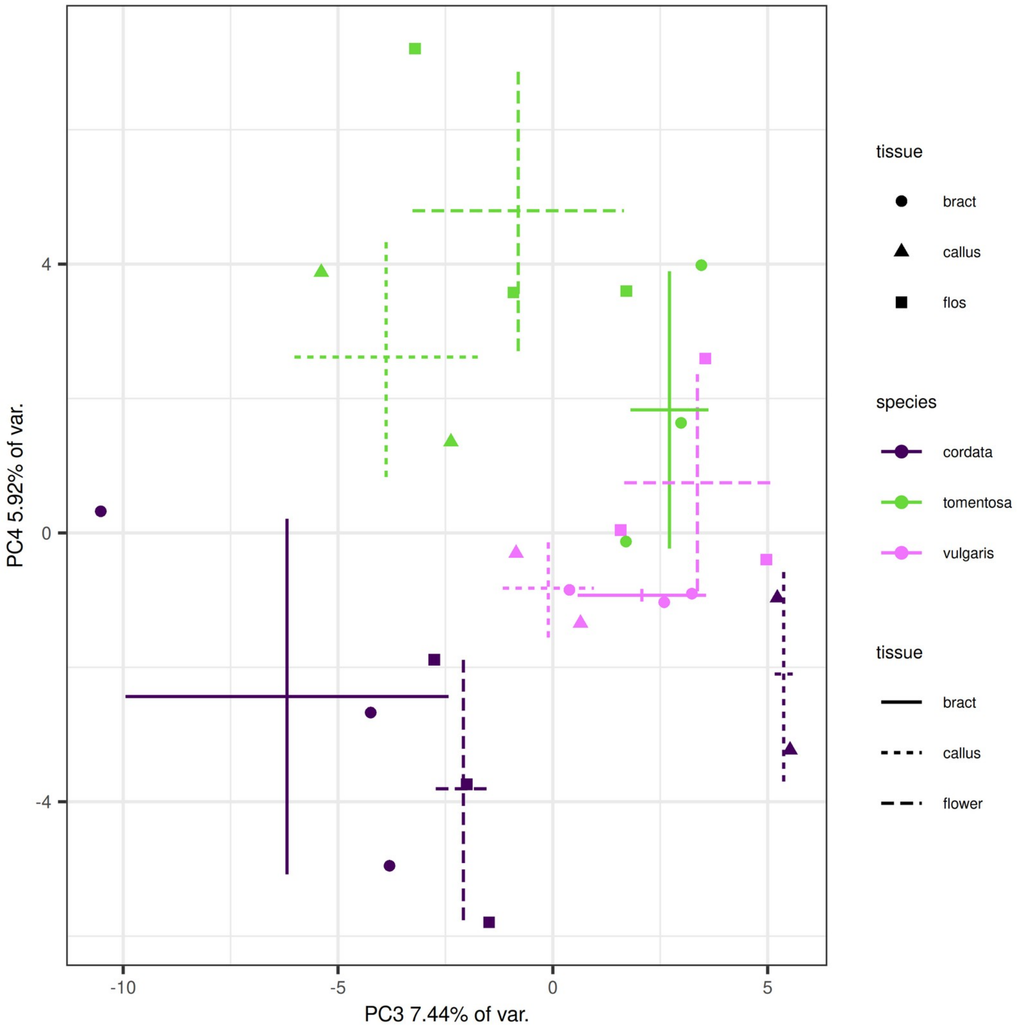

Figure S1. Principal component analysis biplot showing separation of various species of different *Tilia* spp. according to their plant metabolome features. Principal components PC3 and PC4 are plotted. Axes show principal component order, with explained variance. Crosses denote average ± standard deviation for a species–−organ pair (solid line, bract; dashed line, callus; long-dashed line, flower). Point shapes denote tissue type: circle, bract; triangle, callus; square: flower. Color denotes different *Tilia* species: purple, *T. cordata*; green, *T. tomentosa*; magenta, *T. vulgaris*.

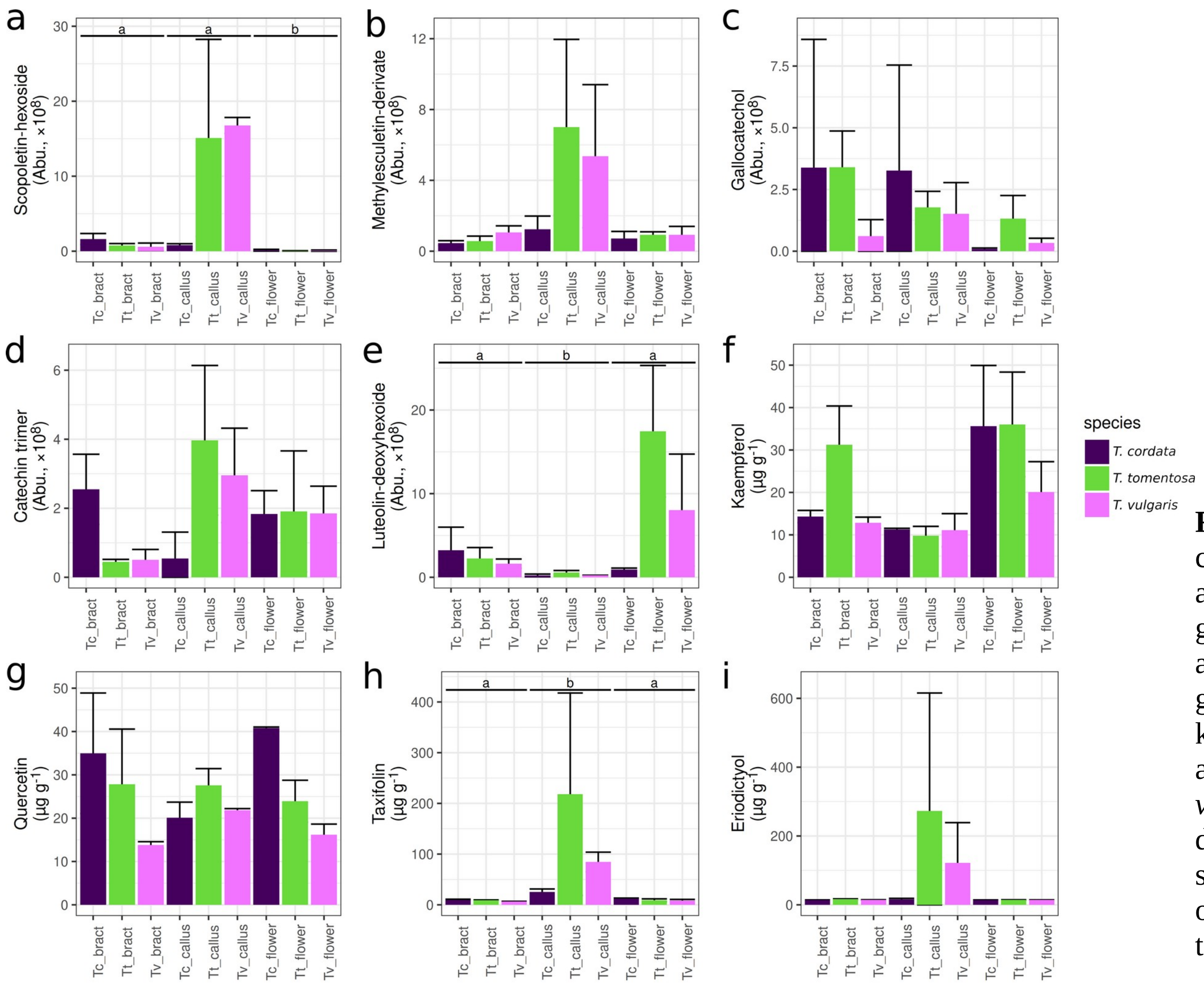

Figure S2. Concentrations or relative abundances of key bioactive constituents from various organs of *Tilia* species. Where an authentic standard was available, µg g^{-1} (dry weight basis) are given. In other subplots, raw abundance data are shown. Subplots: a., scopoletin-O-hexoside; b., methylesculetin derivative; c., gallocatechin; d., catechin trimer; e., luteolin-O-deoxyhexoside; f., kaempferol; g., quercetin; h., taxifolin; i., eriodictyol. Species abbreviations: *Tc*, *Tilia cordata*; *Tt*, *Tilia tomentosa*; *Tv*, *Tilia vulgaris*. Organs not sharing the same letter are significantly different at $p < 0.05$ (Dunn's test, followed by a BY-adjusted, significant Kruskal-Wallis test). Where no letters are present, the organs are not significantly different (BY-adjusted Kruskal-Wallis test).

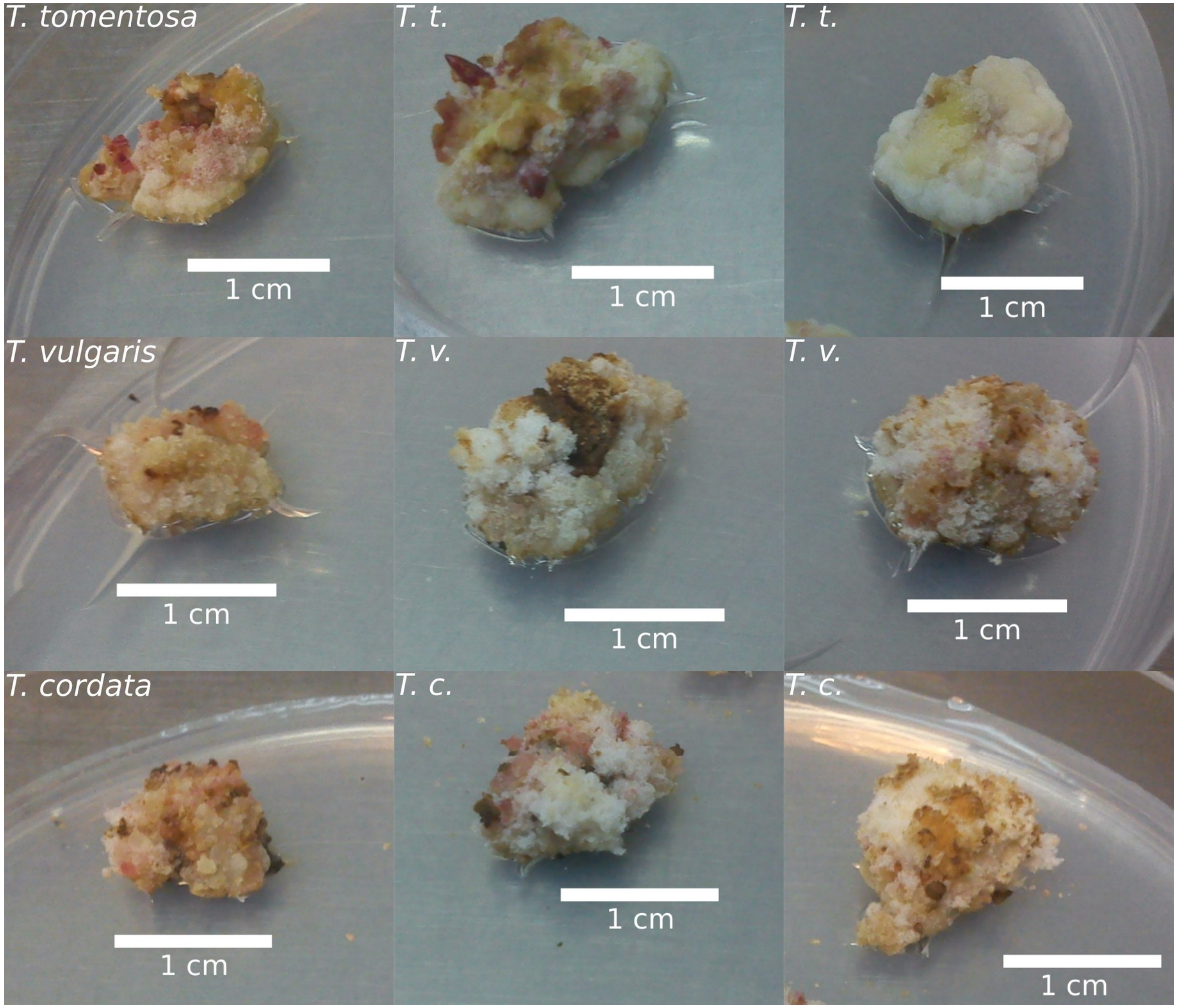

Figure S3. Photos of stable callus cultures of tested *Tilia* species, at the end of their 28-day culture period, before harvesting. Species names are indicated in the subplots in the top left corner.

Table S1. Method performance parameters. Abbreviations: LLOQ, lower limit of quantification; RSD, relative standard deviation; ULOQ, upper limit of quantification.

	Scopoletin	Catechin	Quercetin	Kaempferol	Taxifolin	Eriodictol	Astragalin	Isoquercitrin	Esculin
LLOQ (μg mL^{-1}) [a]	0.01	0.01	0.01	0.01	0.03	0	0.1	0.1	0.01
ULOQ (μg mL^{-1}) [a]	1	25	1	1	1	2.5	25	25	2.5
Equation linearity (R^2) [a]	0.9985	0.9922	0.9979	0.9983	0.9982	0.9969	0.9941	0.9753	0.9985
Equation slope [a]	1.55E+07	2.43E+07	7.22E+07	1.04E+08	3.61E+07	5.38E+07	4.01E+07	2.17E+07	1.44E+08
Equation intercept [a]	1.90E+04	-4.98E+06	-1.82E+06	-2.24E+06	-5.71E+05	-1.90E+06	-1.11E+07	-1.62E+07	-5.09E+06
Intraday repeatability	0.01	0.5	0.01	0.01	0.01	0.01	0.5	1.25	0.13
accuracy, +25% level spike concentration (μg mL^{-1})	133.64%	101.01%	159.07%	149.59%	106.88%	95.31%	112.3%	108.94%	133.79%
accuracy +25% level recovery	0.03	1	0.03	0.01	0.03	0.03	1	2.5	0.25
accuracy, +50% level spike concentration (μg mL^{-1})	91.77%	82.14%	121.39%	112.97%	87.92%	82.53%	92.76%	90.66%	101.05%
accuracy +50% level recovery	0.01	0.01	0.01	0.01	0.03	0	0.1	0.1	0.01

Notes: [a]: data are given for calibration curves used during quantification.

Table S2. [Separate CSV file!]
Features obtained from the untargeted metabolomics approach via LC-ESI-MS. Only the features that passed QC as in section 2.5. are presented.
Column name legend:
mzmed, median of m/z values;
rtmed, median of retention time values (min);
polarity, ESI MS polarity during detection. 1, positive; -1, negative.
pvadj, Kruskal-Wallis test p-value (significant difference among organ types), BY adjusted;
sigtxt, significance for BY adjusted p-values;
cordata_bract - vulgaris_flos, raw abundance values (mean values), Dunn test results (if adjusted Kruskall-Wallis test is significant);
molecularFormula, SIRIUS suggestion for molecular formula;
adduct, NPC.pathway, NPC.pathway.Probability, NPC.superclass, NPC.superclass.Probability, NPC.class, NPC.class.Probability, ClassyFire.most.specific.class, ClassyFire.most.specific.class.Probability, ClassyFire.level.5, ClassyFire.level.5.Probability, ClassyFire.subclass, ClassyFire.subclass.Probability, ClassyFire.class, ClassyFire.class.Probability, ClassyFire.superclass, ClassyFire.superclass.probability : SIRIUS class annotation output with probabilities ranging 0-1;
where: whether a features was found in targeted, untargeted or both fragmentation approaches. If found in both, which yielded a more confident annotation;
name: identification after manual evaluation.

Table S3. Average concentrations of key bioactive constituents from various organs of *Tilia* species. Data are averages of 2-3 replicates and are expressed in µg g^{-1} DW.

Species	Tissue type	Scopoletin	Esculin	Catechin	Quercetin	Kaempferol	Taxifolin	Eriodictol	Astragalin	Isoquercitrin
cordata	bract	3.47	24.44	1,051.23	34.97	14.33	9.26	13.99	824.02	4,471.72
tomentosa	bract	7.28	17.98	1,035.00	27.83	31.26	9.11	16.41	552.69	1,382.96
vulgaris	bract	10.09	25.83	318.15	13.82	12.84	6.60	14.28	340.16	567.68
cordata	callus	22.42	85.36	1,666.61	20.11	11.28	25.20	16.13	141.98	401.58
tomentosa	callus	63.85	201.31	5,936.71	27.57	9.80	218.15	272.98	126.58	440.97
vulgaris	callus	49.19	190.72	3,059.93	21.82	11.10	84.49	122.00	127.56	476.63
cordata	flos	13.89	17.88	124.75	40.79	35.63	12.29	13.99	1,819.00	8,133.86
tomentosa	flos	11.28	15.86	242.68	23.91	36.04	8.95	14.20	4,400.02	6,384.95
vulgaris	flos	16.11	15.89	94.53	16.17	20.11	8.47	14.16	1,183.27	1,672.89

Supplementary, Materials and methods

Section numbering is the same as for the main paper.

4.1. Chemicals. List of vendors for WPM components.

Boric acid (Reanal), calcium chloride (Reanal), calcium nitrate (Reanal), copper-sulfate-pentahydrate (Reanal), disodium-EDTA (Reanal), iron(II)-sulfate (Reanal), magnesium-sulfate (Reanal), manganese sulfate (Reanal), sodium molybdenate (Reanal), potassium dihydrogen phosphate (Reanal), zinc sulfate heptahydrate (Reanal), ammonium nitrate (Reanal), potassium sulfate (Reanal); myo-inozitol (Reanal), nicotinic acid (Reanal), pyridoxine hydrochloride (Carl Roth), thiamine hydrochloride (Carl Roth), saccharose (VWR), agar-agar (VWR), 2,4-dichlorophenoxyacetic acid (2,4-D) (Sigma Aldrich), benzyl-aminopurine (BAP) (Sigma Aldrich).

4.3.1. Sample preparation, drying procedure.

The dried samples were homogenized using a mortar and pestle using liquid N_2. Thereafter, an accurately weighed, approximately 25 mg amount of material was thoroughly mixed with 1 ml MeOH, maintained at room temperature for 10 min and subsequently extracted at 75 °C for 30 min. The mixture was centrifuged at 13,000 rpm for 3 min and the obtained supernatant was stored at −24 °C.

4.3.2. LC-ESI-MS parameters.

The UHPLC system (Dionex Ultimate 3000RS) was coupled with a Thermo Q Exactive Orbitrap mass spectrometer (Thermo Fisher Scientific Inc., Waltham, USA) equipped with an electrospray ionization source (ESI). The HPLC separation was achieved on a Phenomenex Kinetex XB-C18 column (100 mm × 2.1 mm × 2.6 µm). Oven temperature was maintained at 30 °C, and flow rate was 250 µL min−1. Eluent A was water containing 0.1% formic acid and eluent B was acetonitrile (Fisher Scientific, USA) containing 0.1% formic acid. The following gradient elution program was used: 0 min, 2.5% B; 0–2 min, 5% B; 2–7 min, 100% B; 7–8.5 min, 100% B; 8.5-9.5, 2.5% B; 9.5-16, 2.5% B. A 1 µL aliquot of the samples (equivalent to 2.5 µg of plant DW) were injected in every run. The Q Exactive hybrid quadrupole-orbitrap mass spectrometer was operated in either positive or negative ion mode, at the resolution of 35,000 and the scan range was 100–1000 m/z. Additional parameters were: sheath gas flow rate, 32; Aux glas flow rate, 7; Sweep gas flow rate, 0; Spray voltage |kV|, 4; Capillary temperature (C), 320; Aux gas heater temp (C), 60.

4.3.4. Metabolite annotation. MS/MS parameters.

Table S4. Data-dependent MS/MS parameters (ddMS) for targeted LC-MS/MS.

**General**	
Default charge	1
**Full MS**	
Resoultion	35 000
AGC Target	3e6
Maximum IT	100 ms
Scan Range	100 - 1500
**dd-MS^2**	
Resolution	17 500
AGC target	1e5
Maximum IT	50ms
Loop count	5
TopN	5
Isolation window	1.0 m/z
Fixed first mass	-
(N)CE / stepped nce:	30, 50
**dd Settings**	
maximum AGC target	8e3
Intensity threshold	1.6e5
Apex trigger	-
Charge exclusion	2-8, >8
Peptide match	-
Exclude isotopes	on
Dynamic exclusion	2.0s

Table S5. Data-dependent MS/MS parameters (ddMS) for untargeted LC-MS/MS.

**General**	
Default charge	1
**Full MS**	
Resoultion	35 000
AGC Target	3e6
Maximum IT	100 ms
Scan Range	100 - 1500
**dd-MS^2**	
Resolution	17 500
AGC target	2e5
Maximum IT	100ms
Loop count	5
TopN	5
Isolation window	2.0 m/z
Fixed first mass	-
(N)CE / stepped nce:	30
**dd Settings**	
maximum AGC target	8e3
Intensity threshold	8e4
Apex trigger	-
Charge exclusion	2-8, >8
Peptide match	-
Exclude isotopes	on
Dynamic exclusion	2.0s

4.3.5. Quality controlled, untargeted metabolomics

In case of features for which no authentic standards are available, linearity and precision values were estimated from so-called quality control (QC) samples to keep reliably measurable features for downstream analysis [72]. The “intra-study QC” approach was used [72] with parameters already used in our recent study [75].

In particular, a mixture of aliquots from all samples mixed in equal volumes was used, referred to as “intra-study QC”. A serial dilution from 25-fold to concentrated QC was used to assess linearity, two injections were done per concentration. Linearity calculation contained the process blank as a zero. Other QC samples were 10-fold diluted, like the real samples. Samples were injected in a randomized order, after a pre-equilibration block of 10-fold diluted QC samples and the linearity samples. The injection order is given in Table S6. In the main sequence, QC samples were injected for every 6th run. During feature filtering, only those features are used in downstream analysis which (1) showed >0.8 linearity in the set of serially diluted QC samples, (2) showed <0.3 relative standard deviation in QC samples of the main sequence block. The features that passed were subjected to a LOESS readjustment to account for local inhomogeneities of sensitivity along the sequence [75], expressing values as fold changes versus QC.

Table S6. XCMS Online automated peak search parameters.

**Feature detection**	
method	centWave
ppm	2.5
minimum peak width	2.5
maximum peak width	25
mzdiff	0.01
Signal/Noise threshold	10
Integration method	1
prefilter peaks	3
prefilter intensity	5000
Noise Filter	1000
**Retention time correction**	
method	obiwarp
profStep	1
**Alignment**	

bw	5
minfrac	0.5
mzwid	0.02
minsamp	1
max	100

Table S7. Injection order for untargeted metabolomics

**Block**	**Sample**
wash	wash
blank	solventblank_i1
blank	solventblank_i2
blank	processblank_i1
blank	processblank_i2
sst	sst
calibration_curve	cc1_0_01_i1
calibration_curve	cc1_0_025_i1
calibration_curve	cc1_0_1_i1
calibration_curve	cc1_0_25_i1
calibration_curve	cc1_1_i1
calibration_curve	cc1_2_5_i1
calibration_curve	cc1_10_i1
calibration_curve	cc2_0_01_i1
calibration_curve	cc2_0_025_i1
calibration_curve	cc2_0_1_i1
calibration_curve	cc2_0_25_i1
calibration_curve	cc2_1_i1
calibration_curve	cc2_2_5_i1
calibration_curve	cc2_10_i1
pre-equilibration	qcpre_i01
pre-equilibration	qcpre_i02
qc_linearity	qc_25xd_i1
qc_linearity	qc_25xd_i2
qc_linearity	qc_10xd_i1
qc_linearity	qc_10xd_i2
qc_linearity	qc_05xd_i1
qc_linearity	qc_05xd_i2
qc_linearity	qc_01xd_i1

qc_linearity	qc_01xd_i2
pre-equilibration	qcpre_i03
pre-equilibration	qcpre_i04
qc_accuracy	qca_1_i1
qc_accuracy	qca_1_i2
qc_accuracy	qca_1_i3
qc_accuracy	qca_2_5_i1
qc_accuracy	qca_2_5_i2
qc_accuracy	qca_2_5_i3
qc_accuracy	qca_10_i1
qc_accuracy	qca_10_i2
qc_accuracy	qca_10_i3
pre-equilibration	qcpre_i05
pre-equilibration	qcpre_i06
pre-equilibration	qcpre_i07
real_block_w_qcs	qc_rei_i1
real_block_w_qcs	QC
real_block_w_qcs	cordata_flos_2
real_block_w_qcs	tomentosa_flos_2
real_block_w_qcs	vulgaris_bract_1
real_block_w_qcs	vulgaris_flos_3
real_block_w_qcs	QC
real_block_w_qcs	vulgaris_flos_1
real_block_w_qcs	tomentosa_callus_1
real_block_w_qcs	qc_rei_i2
real_block_w_qcs	tomentosa_flos_3
real_block_w_qcs	vulgaris_flos_2
real_block_w_qcs	QC
real_block_w_qcs	tomentosa_bract_3
real_block_w_qcs	cordata_flos_3

real_block_w_qcs	cordata_callus_2
real_block_w_qcs	tomentosa_bract_1
real_block_w_qcs	QC
real_block_w_qcs	qc_rei_i3
real_block_w_qcs	vulgaris_callus_2
real_block_w_qcs	cordata_flos_1
real_block_w_qcs	tomentosa_flos_1
real_block_w_qcs	cordata_bract_2
real_block_w_qcs	QC
real_block_w_qcs	cordata_bract_3
real_block_w_qcs	vulgaris_bract_3
real_block_w_qcs	vulgaris_callus_1
real_block_w_qcs	cordata_bract_1
real_block_w_qcs	qc_rei_i4
real_block_w_qcs	QC
real_block_w_qcs	vulgaris_bract_2
real_block_w_qcs	tomentosa_callus_2
real_block_w_qcs	tomentosa_bract_2
real_block_w_qcs	cordata_callus_1
real_block_w_qcs	QC
real_block_w_qcs	qc_rei_i5
qualitative_block	aif_neg
qualitative_block	ddms_neg_mzrange_1
qualitative_block	ddms_neg_mzrange_2
qualitative_block	ddms_neg_mzrange_3
qualitative_block	ddms_neg_mzrange_4
qualitative_block	ddms_neg_mzrange_5
qualitative_block	ddms_neg_mzrange_6
qualitative_block	ddms_neg_mzrange_7
qualitative_block	ddms_neg_mzrange_8

qualitative_block	ddms_neg_mzrange_9
qualitative_block	aif_pos
qualitative_block	ddms_pos_mzrange_1
qualitative_block	ddms_pos_mzrange_2
qualitative_block	ddms_pos_mzrange_3
qualitative_block	ddms_pos_mzrange_4
qualitative_block	ddms_pos_mzrange_5
qualitative_block	ddms_pos_mzrange_6
qualitative_block	ddms_pos_mzrange_7
qualitative_block	ddms_pos_mzrange_8
qualitative_block	ddms_pos_mzrange_9
wash	wash_1
calibration_curve	cc1_0_01_i2
calibration_curve	cc1_0_025_i2
calibration_curve	cc1_0_1_i2
calibration_curve	cc1_0_25_i2
calibration_curve	cc1_1_i2
calibration_curve	cc1_2_5_i2
calibration_curve	cc1_10_i2
calibration_curve	cc2_0_01_i2
calibration_curve	cc2_0_025_i2
calibration_curve	cc2_0_1_i2
calibration_curve	cc2_0_25_i2
calibration_curve	cc2_1_i2
calibration_curve	cc2_2_5_i2
calibration_curve	cc2_10_i2

References

72. Kirwan, J.A.; Gika, H.; Beger, R.D.; Bearden, D.; Dunn, W.B.; Goodacre, R.; Theodoridis, G.; Witting, M.; Yu, L.-R.; Wilson, I.D.; et al. Quality Assurance and Quality Control Reporting in Untargeted Metabolic Phenotyping: mQACC Recommendations for Analytical Quality Management. Metabolomics 2022, 18, 70. https://doi.org/10.1007/s11306-022-01926-3.

75. Gonda, S.; Szűcs, Z.; Plaszkó, T.; Cziáky, Z.; Kiss-Szikszai, A.; Sinka, D.; Bácskay, I.; Vasas, G. Quality-Controlled LC-ESI-MS Food Metabolomics of Fenugreek (Trigonella foenum-Graecum) Sprouts: Insights into Changes in Primary and Specialized Metabolites. Food Res. Int. 2023, 164, 112347. https://doi.org/10.1016/j.foodres.2022.112347.
